# Supplementary material for: Cryo-EM structures provide insight into how E. coli F1Fo ATP synthase accommodates symmetry mismatch
Source: Nat Commun. 2020 May 26;11:2615. doi: 10.1038/s41467-020-16387-2 (PMC7251095; doi:10.1038/s41467-020-16387-2)
Supplement: Supplementary file 1 — Supplementary Information [file 41467_2020_16387_MOESM1_ESM.pdf]

SUPPLEMENTARY INFORMATION

Cryo-EM structures provide insight into how *E. coli* F_1F_o ATP synthase accommodates symmetry mismatch

Meghna Sobti et al.

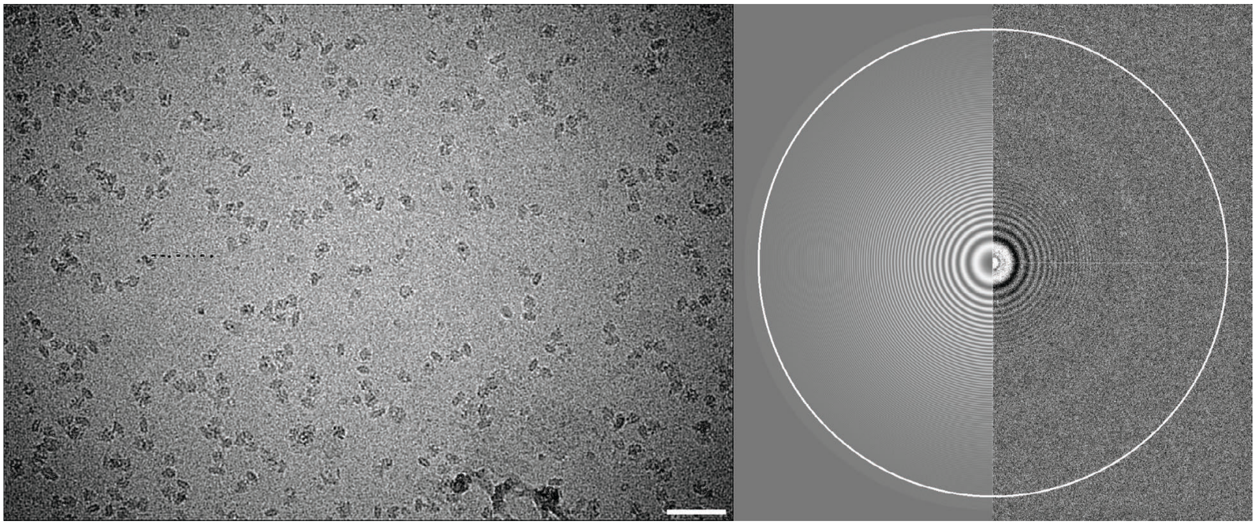

Supplementary Figure 1 Cryo-EM micrograph and power spectrum (**a**) Representative micrograph showing *E. coli* ATP synthase particles (white scale bar is equivalent to 50 nm) and (**b**) Gctf output of same micrograph. The data was taken on a distinct sample, we have imaged *E. coli* ATP synthase many times[1,2] and taken full cryo-EM datasets ~10 times. The micrograph shown was chosen to show clear ATP synthase particles (high defocus) and is devoid of ice contamination and particle aggregation.

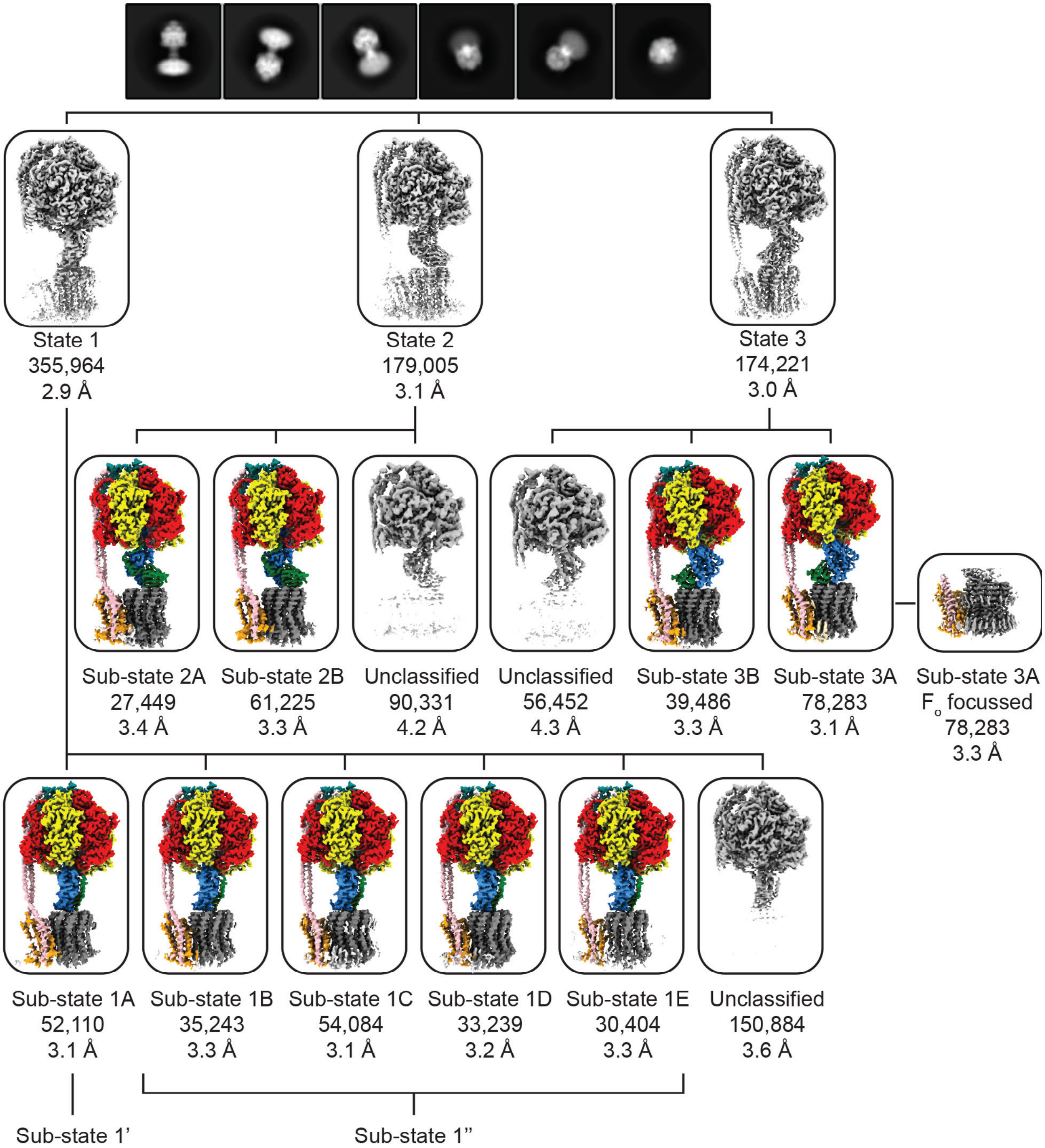

Supplementary Figure 2 Flowchart describing the classification of particles into distinct rotary conformations.

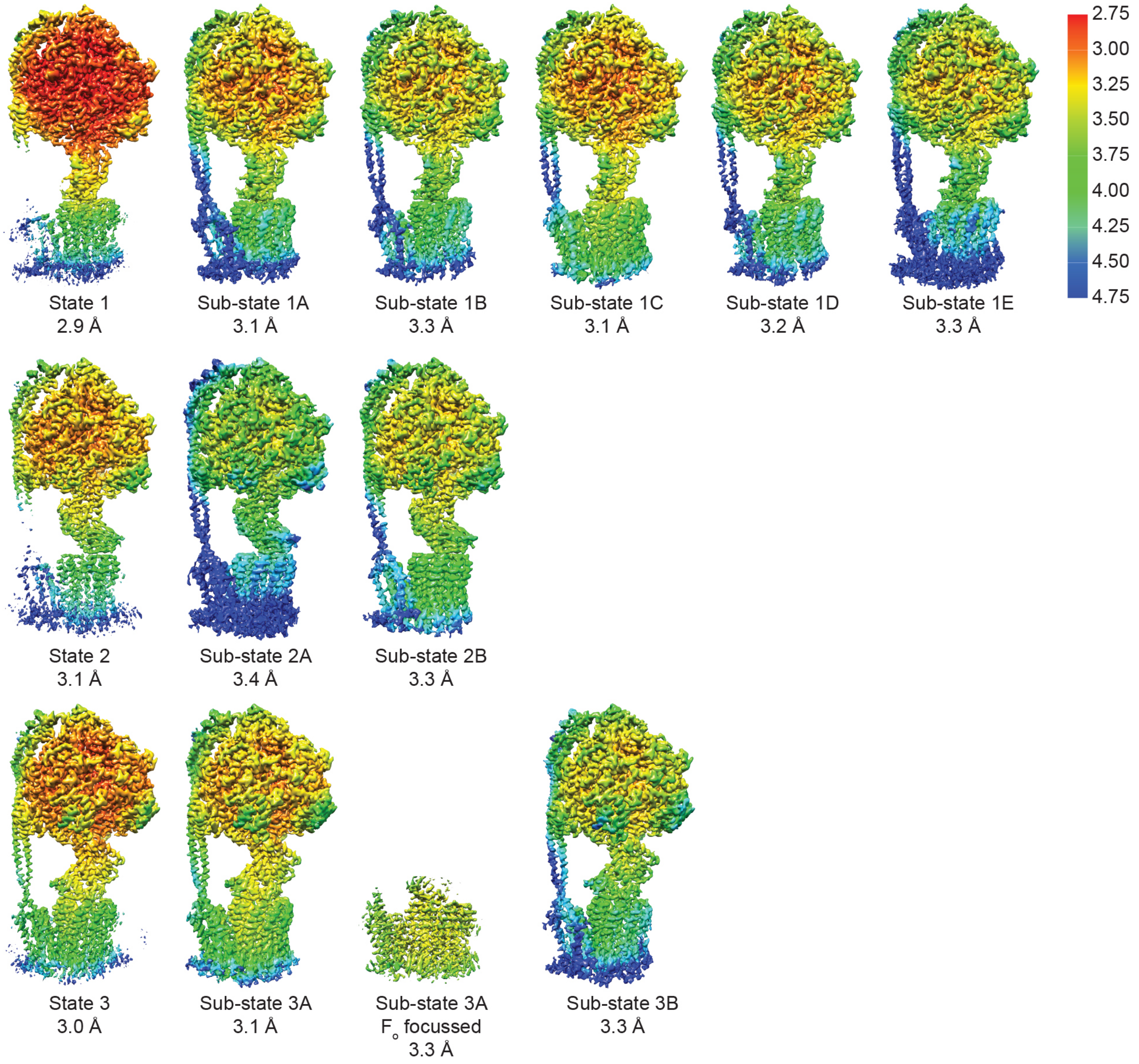

Supplementary Figure 3 Local resolution plots of each state and sub-state described in this study. Local resolution implemented in Relion[3] and output displayed in Chimera with color key in top right.

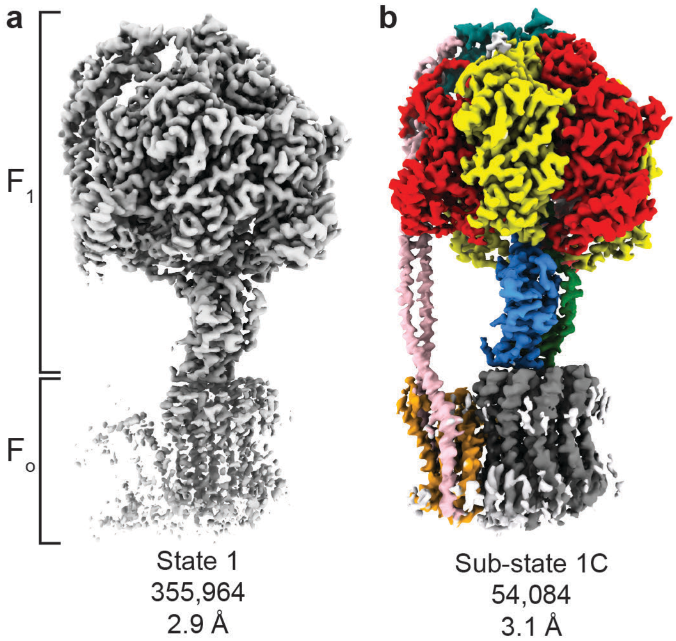

Supplementary Figure 4 Focused sub-classification reveals increased detail in the F_o region.

(**a**) State 1 and (**b**) Sub-state 1C

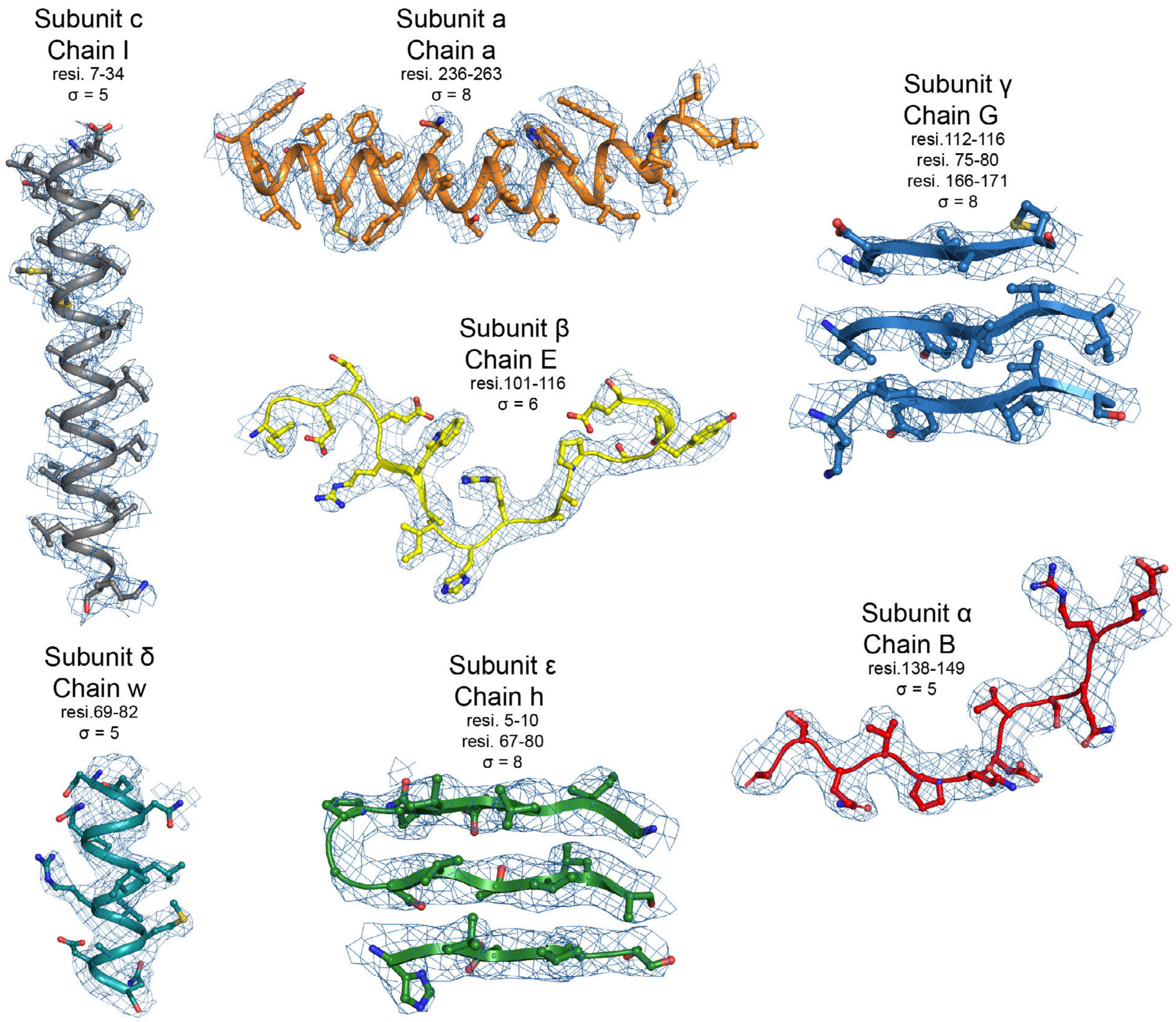

Supplementary Figure 5 Cryo-EM density of regions within Sub-state 3A. Representative samples of sections showing the modelled coordinates in the cryo-EM density for given subunits and their corresponding chain IDs. Map threshold given in σ.

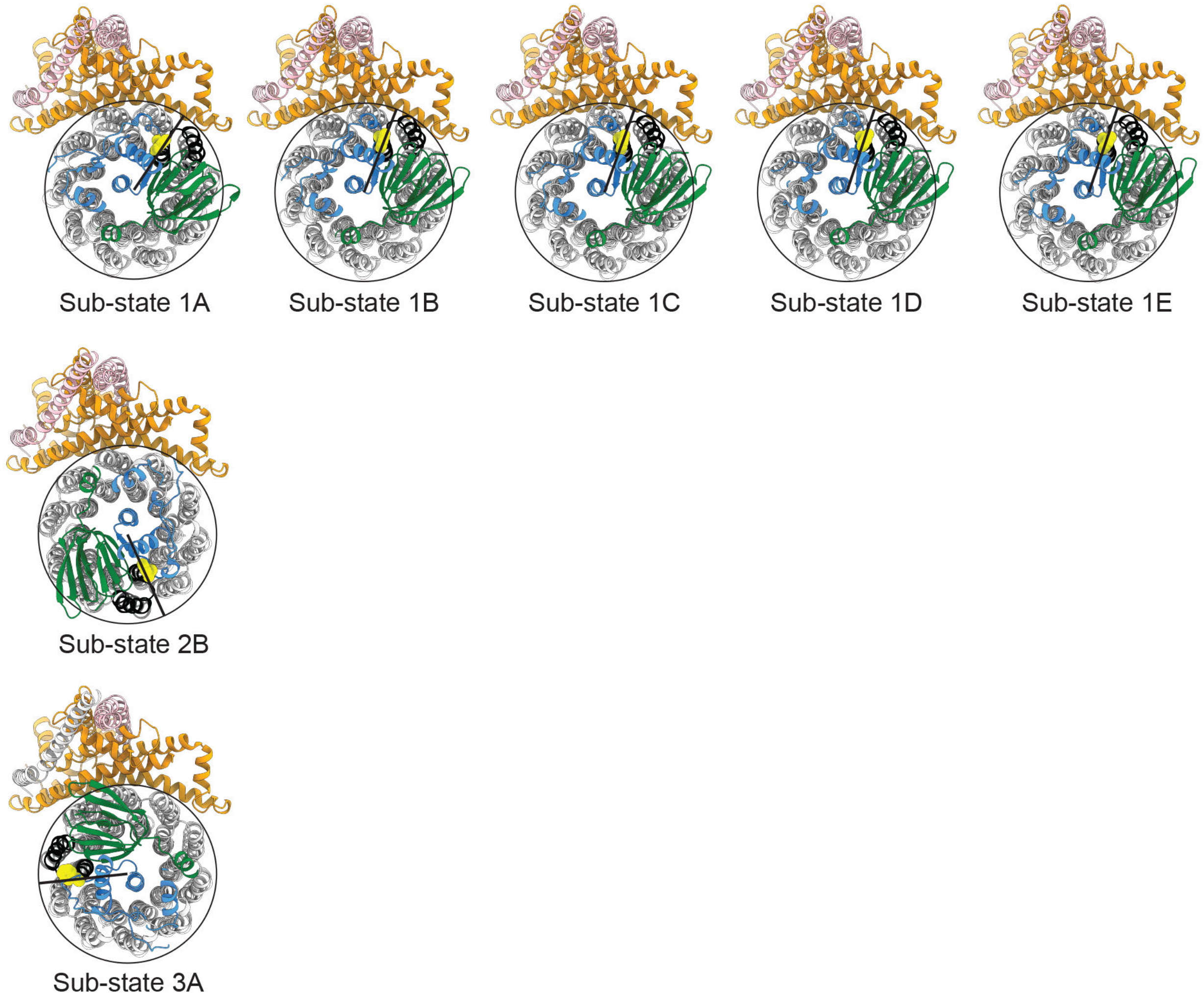

Supplementary Figure 6 Identification of relative rotor position between states/sub-states. The relative position of subunit γ was used to assign the c-ring rotational position. Each atomic model is aligned to the F_o stator region and viewed from the F_1-ATPase. The c subunit (colored black) below γTrp203 (colored yellow) is assigned a letter in the atomic model. This can then be used to compare the relative position of the c-ring between the sub-states across this study.

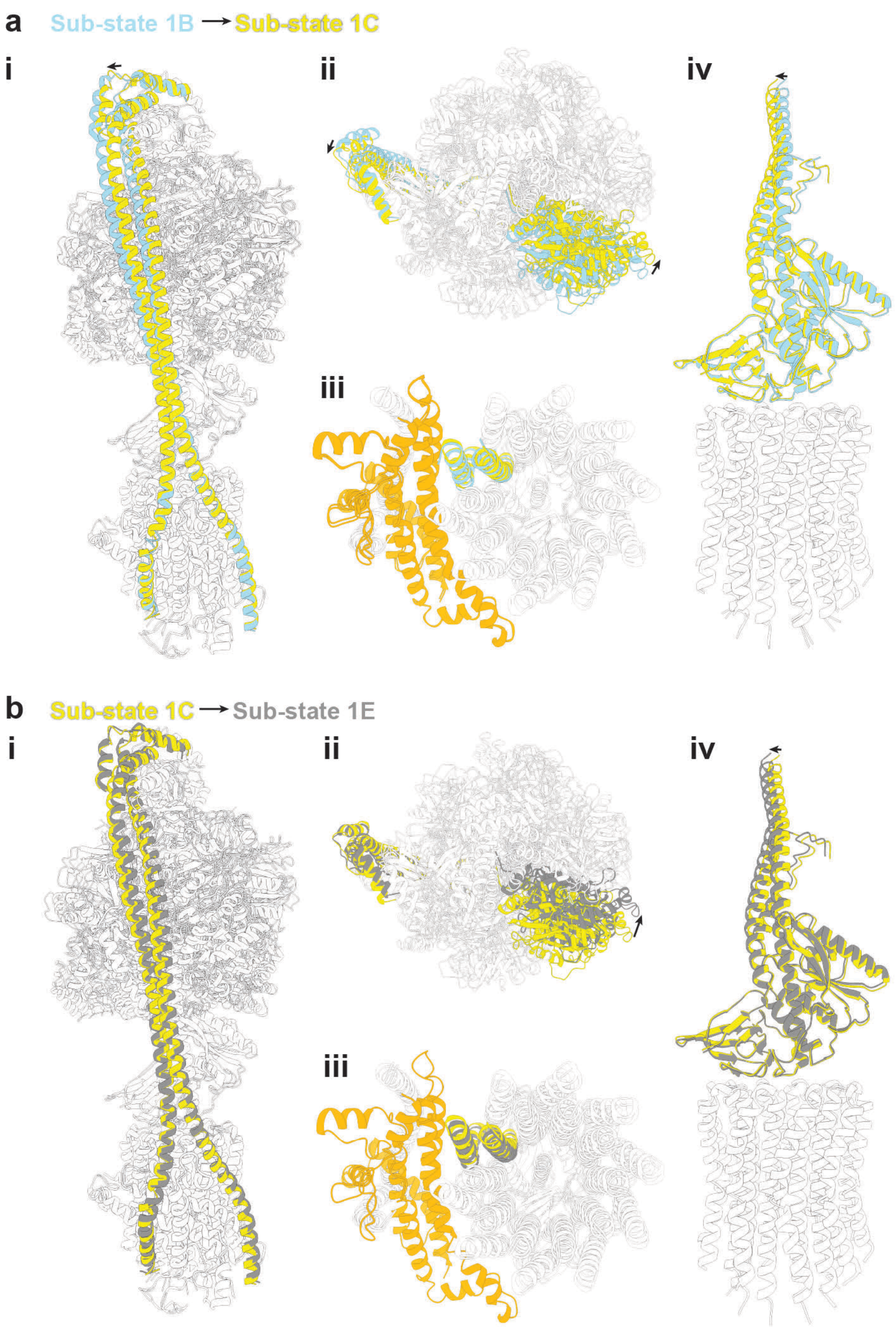

Supplementary Figure 7 Comparison of Sub-states 1B-E (analogous to Figure 2 which compares Sub-states 1A and 1E). These movements do not allow a sub-step in the F_o motor and instead facilitate torsional flexing of the complex. (**a**) Compares Sub-state 1B with Sub-state 1C and (**b**) compares Sub-state 1C with Sub-state 1E. (**i**) Molecular models superposed on stator

subunit a viewed from the side. The peripheral stalk of Sub-state 1B is colored in cyan, Sub-state 1C colored in yellow and Sub-state 1E in grey. (**ii**) Superposition on stator subunit a as viewed from above highlights rotational movement of the of the $\alpha_3\beta_3\gamma\varepsilon c_{10}$ relative to the stator (black arrows). The peripheral stalk and the same α are colored in cyan for Sub-state 1B, yellow for Sub-state 1C and grey for Sub-state 1E. (**iii**) Superposition on stator subunit a as viewed from below shows the ~36° rotation of $\alpha_3\beta_3\gamma\varepsilon c_{10}$ relative to the a subunit (colored in orange), with the same c subunit colored cyan for Sub-state 1B, yellow for Sub-state 1C and grey for Sub-state 1E. (**iv**) Molecular models of the central rotor superposed on the c-ring highlight a small movement at the interface between the stalk and c-ring. The γ and ε subunits of Sub-state 1B are colored in cyan, Sub-state 1C colored in yellow and Sub-state 1E in grey.

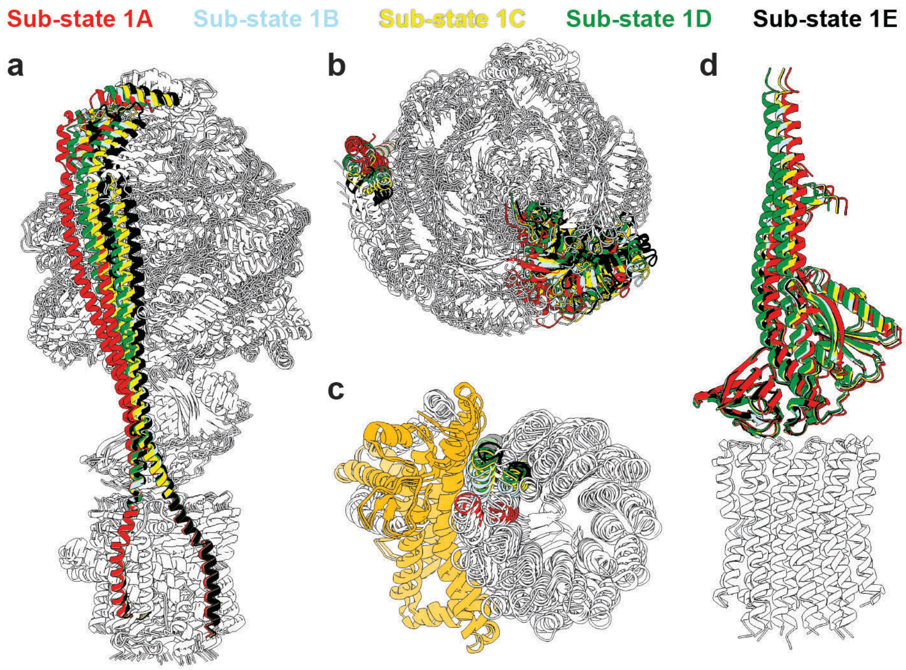

Supplementary Figure 8 Comparison of Sub-states 1A-E. Figure to compare all sub-states of State 1 in a single figure. Views are as in Supplementary Figure 7, and sub-states colored as depicted at the top of the figure.

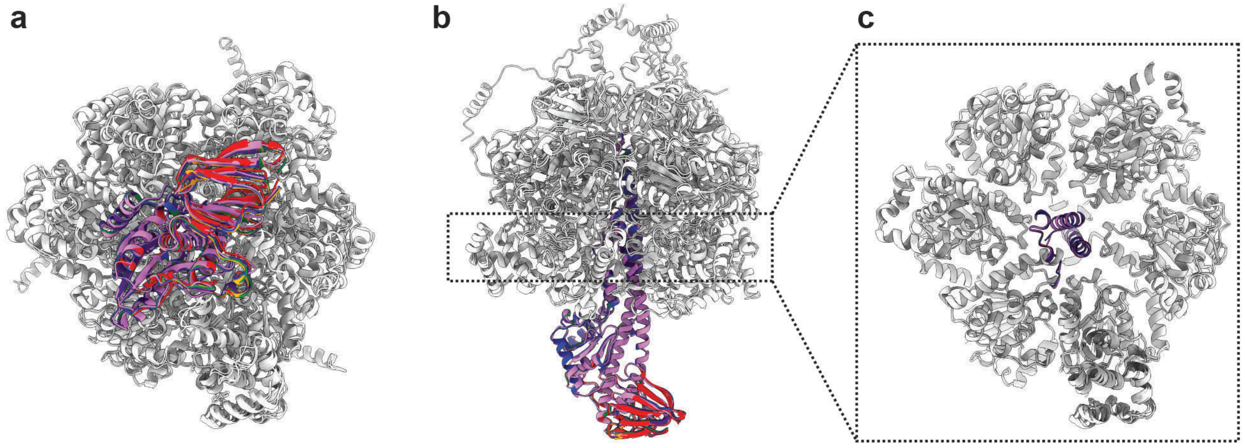

Supplementary Figure 9 The F_1-ATPase is in the same rotational state in all Sub-states. Sub-state 1A in red, 1B in orange, 1C yellow, 1D green, 1E blue, 2B indigo and 3A violet. (**a**) viewed from underneath. (**b**) viewed from the side. (**c**) slice through the F_1-ATPase.

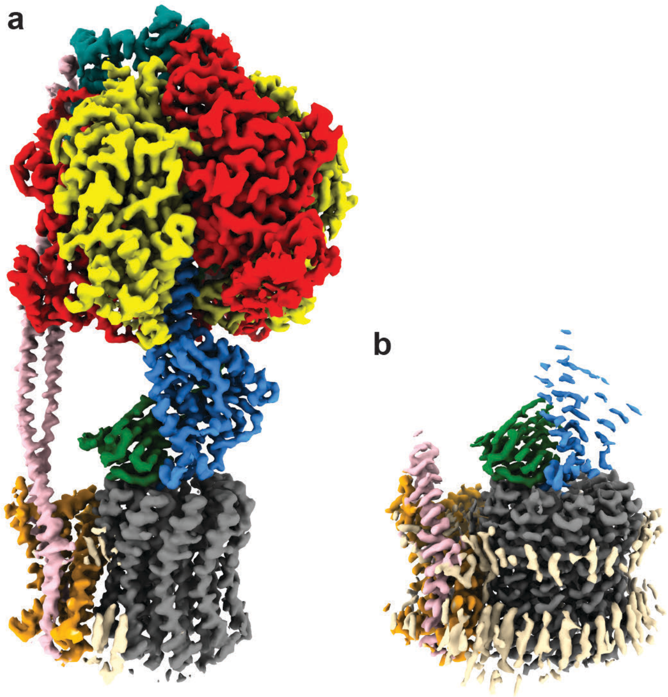

Supplementary Figure 10 Comparison of Sub-state 3A F_o unfocused and focused maps. (**a**) Unfocussed map showing entire F_1F_o complex. (**b**) F_o focused map aligned to just the F_o region shows much clearer density, particularly for the F_o stator (pink and orange subunits) as well as potential lipids (wheat).

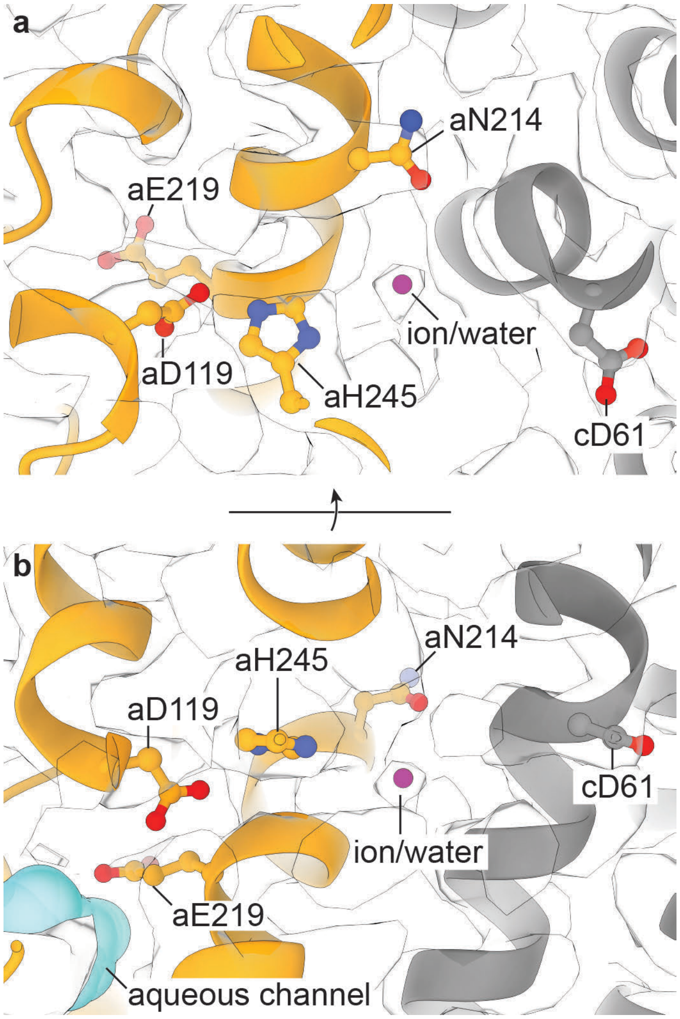

Supplementary Figure 11 Local density protonation channel of *E. coli* ATP synthase. Residues aGlu219, aAsp119, aHis245 and aAsn214 make a continuous line of residues that could facilitate proton translocation between the aqueous channel (blue spheres) and the rotation path of the proton transferring cAsp61. Density consistent with a ion/water molecule is present between residues aHis245 and aAsn214 (highlighted with magenta sphere). (**a**) viewed from the periplasm and (**b**) viewed from within the membrane. The map for Sub-state 3A F_o focused was used to make this figure.

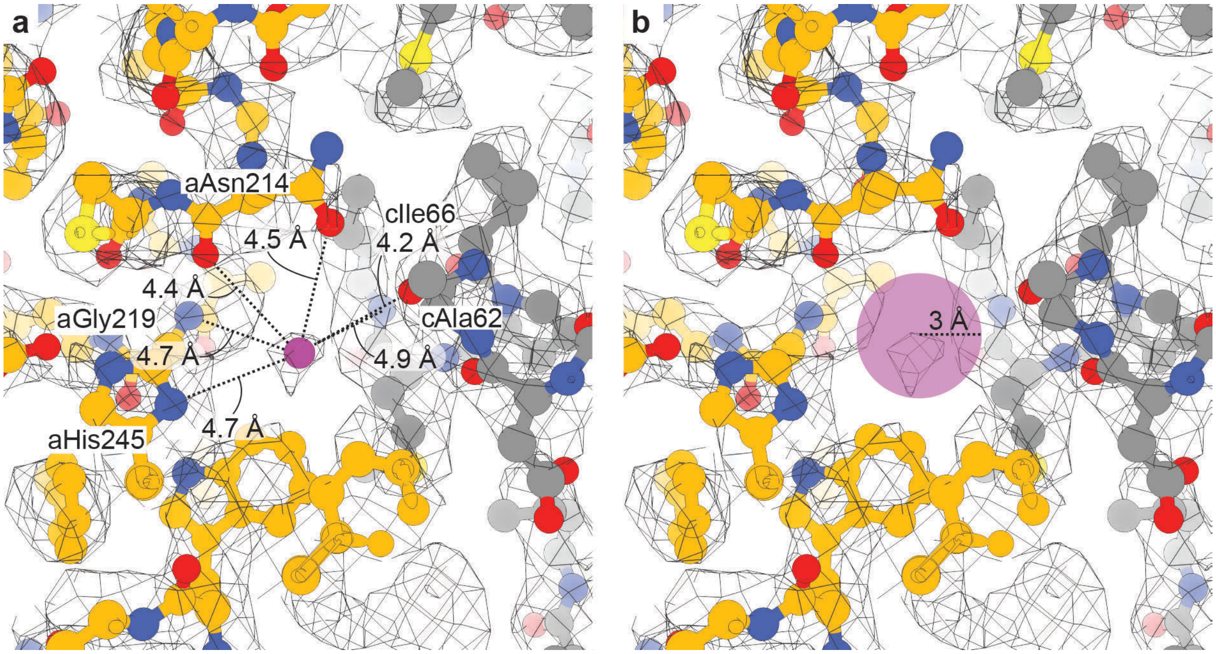

Supplementary Figure 12 Distances around potential ion/water site. (**a**) The distances to polar atoms around the putative ion/water site are too large to be directly hydrogen bonded to a single water molecule. Distances are measured from the ion/water site (magenta) to polar atoms within 5 Å. (**b**) The pocket in which the density resides has a van der Waals radius of at least 3 Å (magenta sphere). The map for Sub-state 3A F_o focused was used to make this figure.

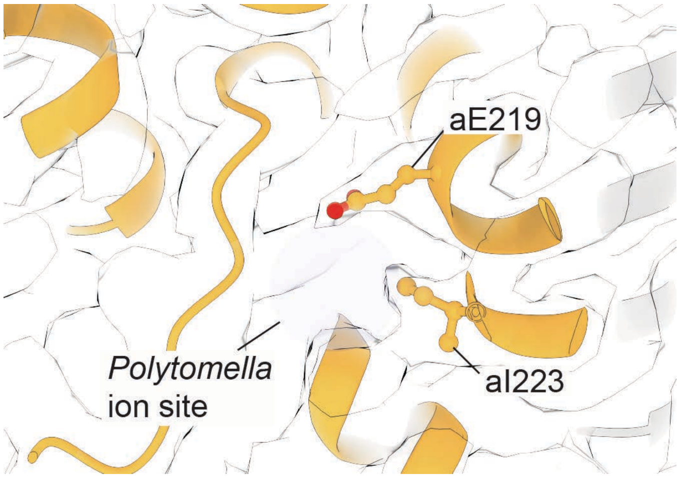

Supplementary Figure 13 Local density around the putative metal ion binding site identified in *Polytomella* ATP synthase. No non-protein density could be observed in the putative metal ion binding site (highlighted with transparent blue circle and labelled) adjacent to residues aGlu219 and aIle223. The map for Sub-state 3A F_o focused was used to make this figure.

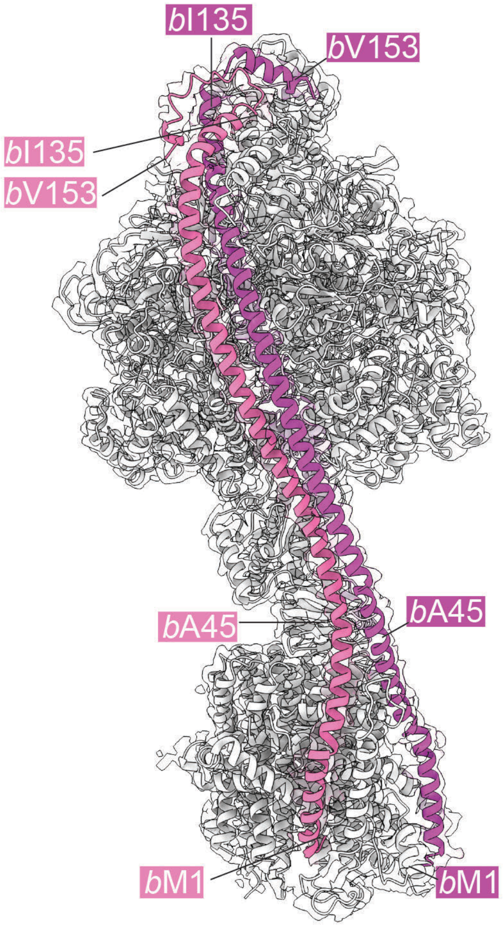

Supplementary Figure 14 The peripheral stalk of *E. coli* F_1 F_o ATP synthase. The two b subunits of the peripheral stalk can be broken into three parts: the N-terminal section (b1-b45) adjacent to the a subunit, the middle right-handed coiled coil (b46-b135) and the C-terminal cap (b136-b154) that attaches to the F_1-ATPase. The map for Sub-state 3A was used to make this figure.

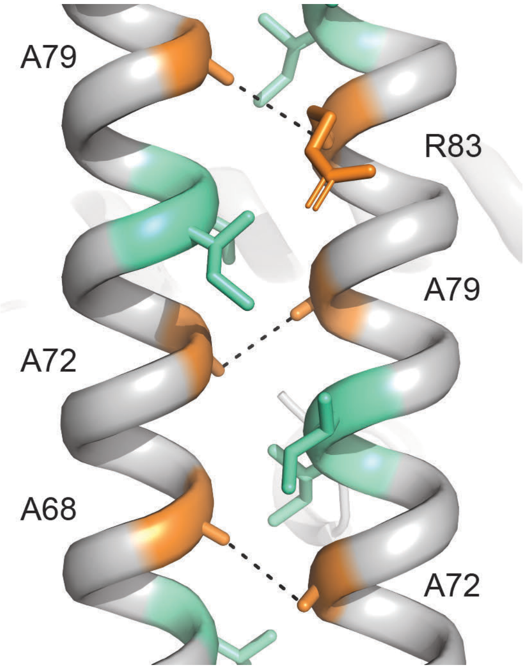

Supplementary Figure 15 Crosslinking studies on the peripheral stalk of *E. coli* F_1F_o ATP synthase fit the cryo-EM structure. Model of a section of the *E. coli* F_1F_o ATP synthase peripheral stalk shows that crosslinks (dotted black lines) previously designed[17] between residues describe the helical orientation and register of the helices.

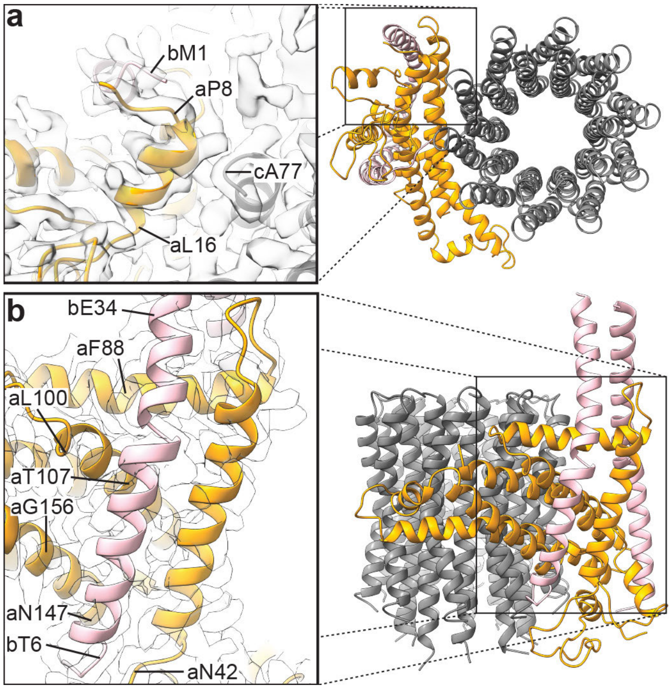

Supplementary Figure 16 Interactions of subunit a and b of the F_o stator. (**a**) View from below, the N-terminus of subunit a from a short helix (orange: aP8-L16) which interacts with the N-terminus of subunit b (pink) and c (grey). (**b**) View from the side, the N-terminal helix of the other b subunit (pink) interacts with residues N42-F88, L100-T107 and N147-G156 of the a subunit (orange). The map for Sub-state 3A F_o focused was used to make this figure.

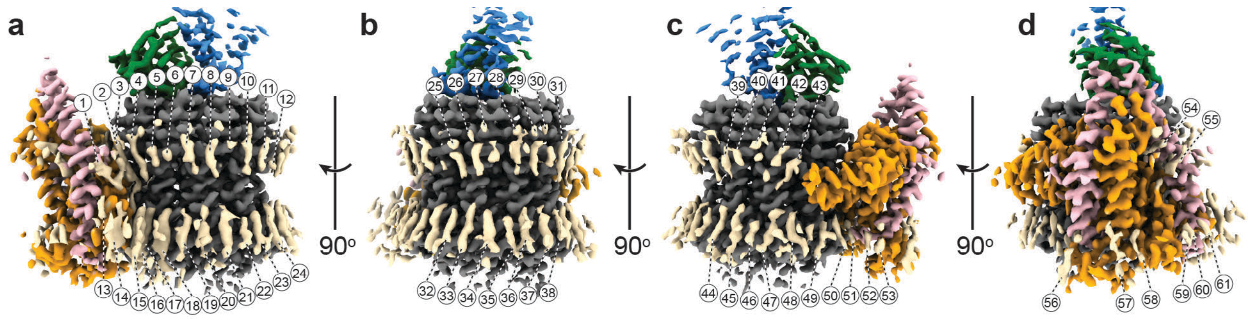

Supplementary Figure 17 Lipids surround the F_o motor. At least 61 lipid tail-like densities can be seen around the F_o motor. (**a**), (**b**), (**c**) and (**d**) are rotated 90° relative to each other as indicated. The map for Sub-state 3A F_o focused was used to make this figure.

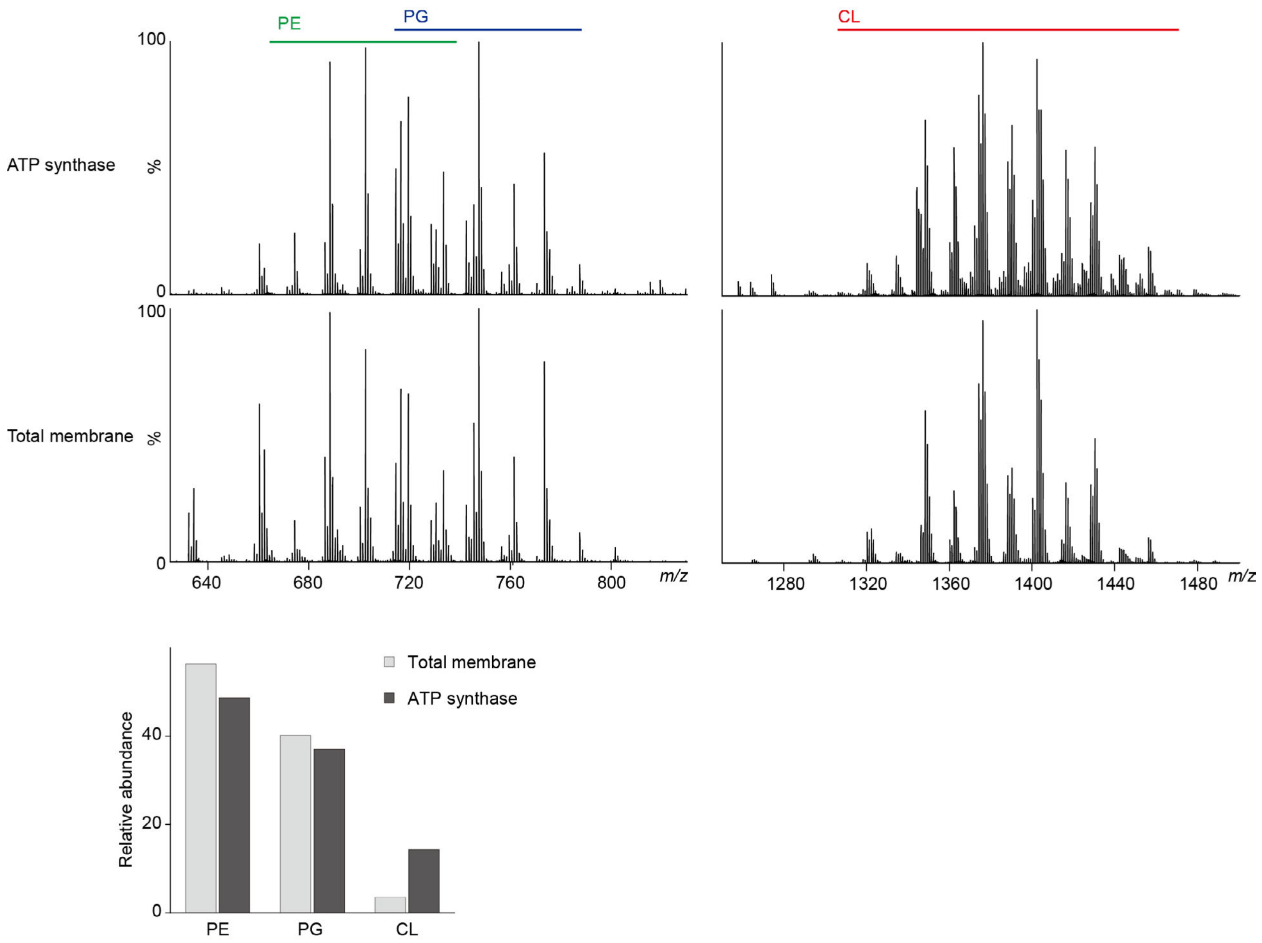

Supplementary Figure 18 Lipid mass spectrometry Phosphatidylethanolamine (PE), phosphatidylglycerol (PG) and cardiolipin (CL) were identified as co-purified lipids in ATP synthase sample. The abundance of each lipid was manually calculated and plotted as bar graph[4]. Comparing to *E. coli* total membrane lipids (100500, Avanti Polar Lipids), CL content in ATP synthase sample was enriched.

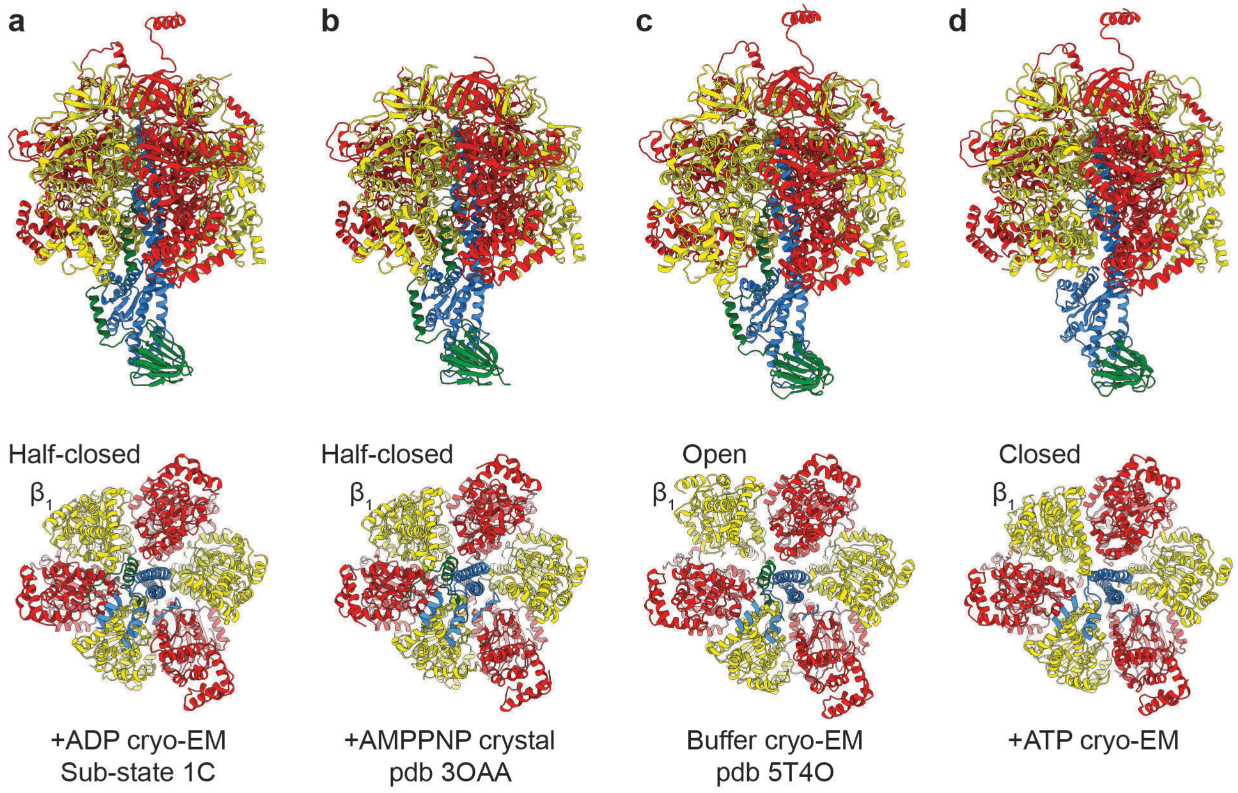

Supplementary Figure 19 All known *E. coli* F_1-ATPase structures. Comparison of all published *E. coli* F_1-ATPase structures. (**a**) Sub-state 1C of this study (solved in the presence of 10 mM MgADP) and (**b**) the crystal structure solved in the presence of 1 mM AMP PNP[5] show essentially the same conformation, with the β_1 subunit in a half-closed conformation, contacting with the ε C-terminal helix. (**c**) When imaged in buffer without nucleotide the β_1 subunit opens[1]. (**d**) When imaged after incubation with 10 mM MgATP the β_1 subunit closes[2].

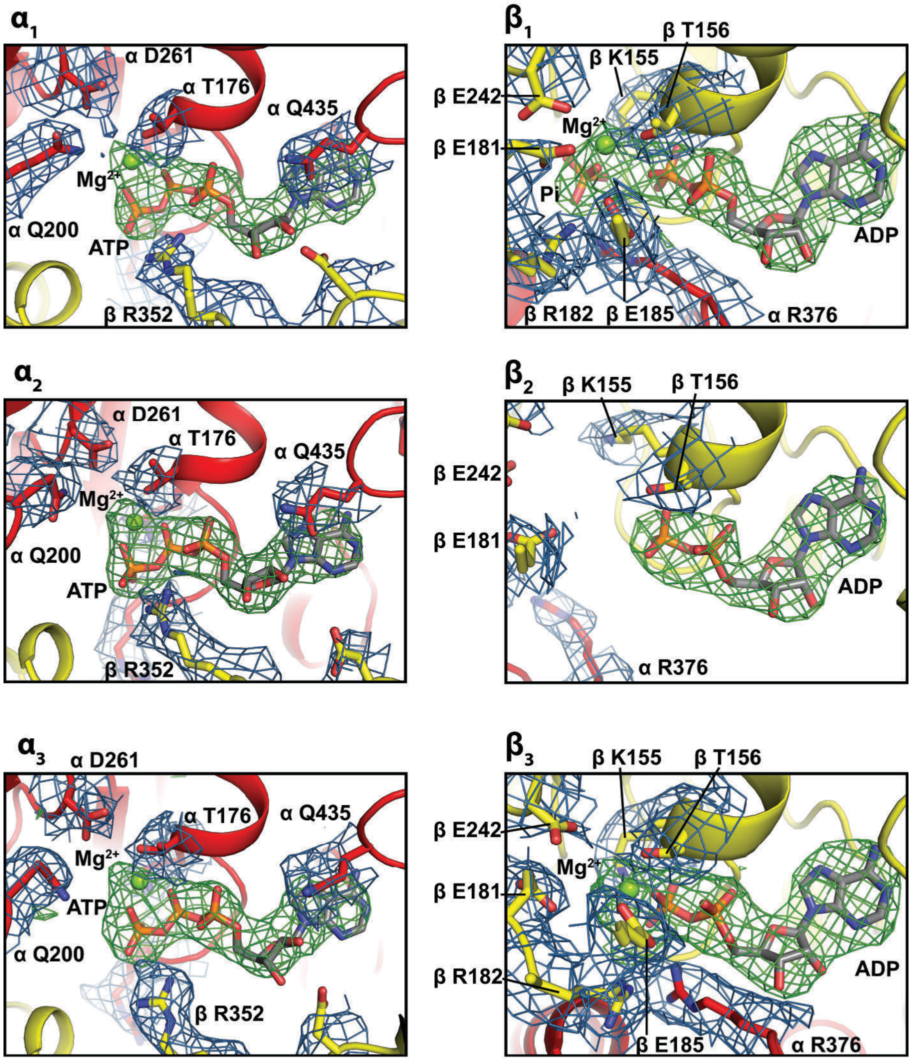

Supplementary Figure 20 F_1-ATPase nucleotide assignment Atomic models (shown as cartoon and stick representation), cryo-EM density maps for residues coordinating the six nucleotide binding sites (blue mesh) and the difference density map of the nucleotides (green mesh) in the F_1-ATPase of Sub-state 3A. Subunits a in red and b in yellow, nucleotide shown in grey, with CPK coloring for atoms. Difference density calculated using phenix.real_space_diff_map[6].

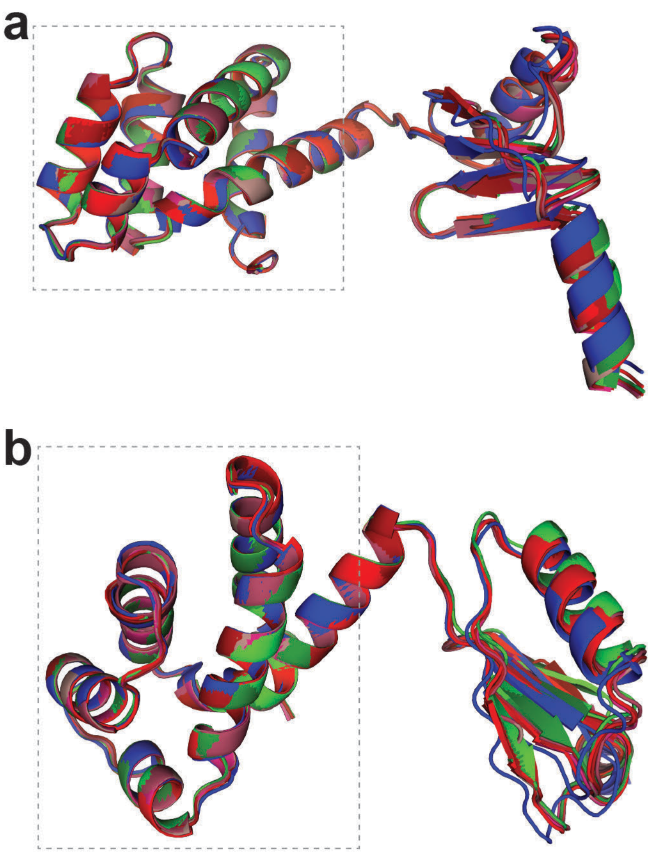

Supplementary Figure 21 Comparison of the δ subunits. The structure of subunit δ is very similar between all states with the r.m.s.d between states being less than 1 Å. Subunit δs were superposed onto the boxed region shown (N-terminal helical bundle) to identify any movement in the suggested hinge region. State 1 in shades of red, State 2 in shades of green and State 3 in shades of blue.

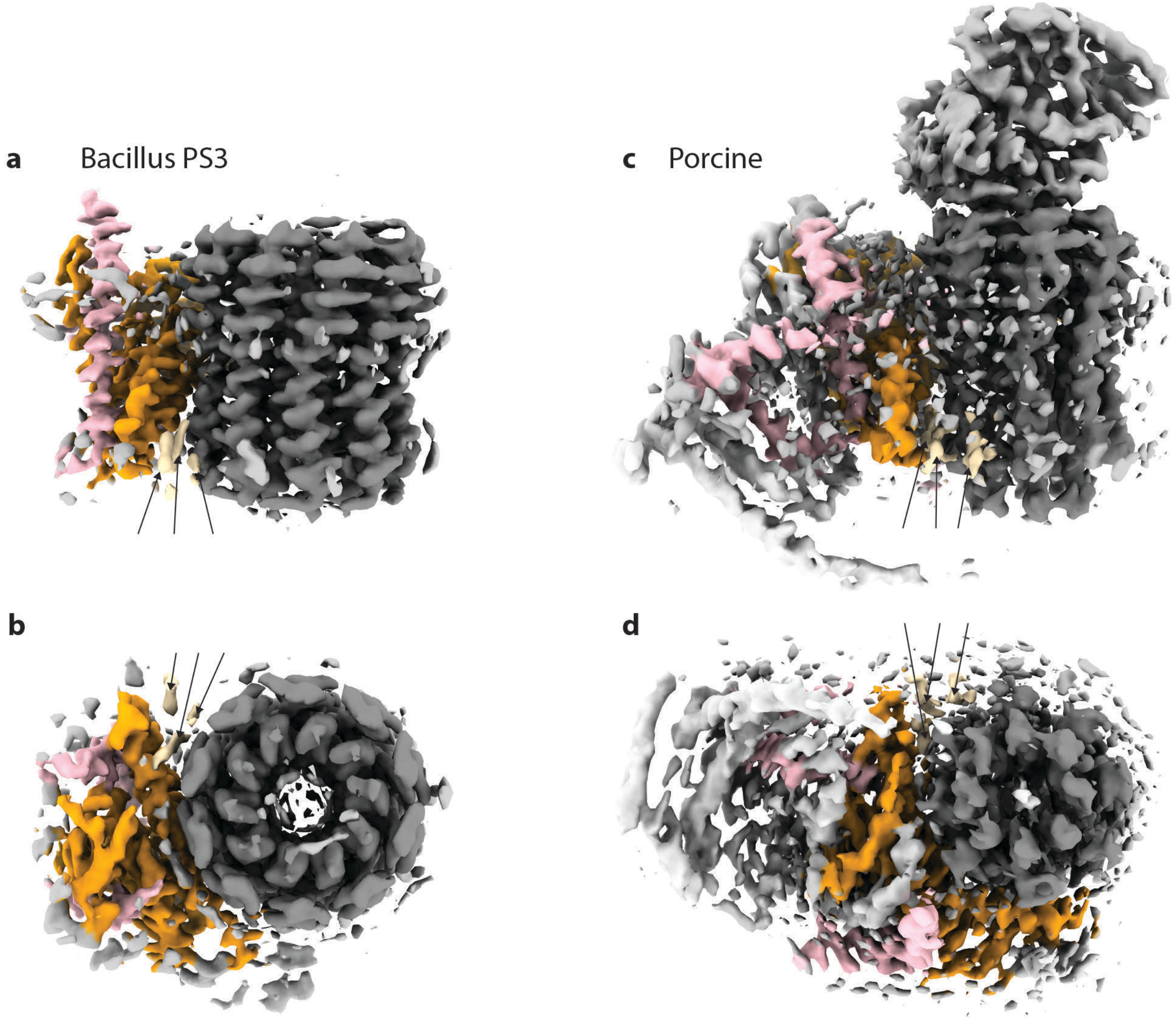

Supplementary Figure 22 *Bacillus sp.* PS3 and porcine ATP synthase also contain lipid-like density. Cryo-EM maps of (**a** and **b**) *Bacillus sp*. PS3[7] (emd9327) and (**c** and **d**) porcine ATP synthase[8] (emd0668) shown as surface. Side views (**a** and **c**) and bottom views (**b** and **d**). Potential lipid density colored wheat and highlighted with black arrows.

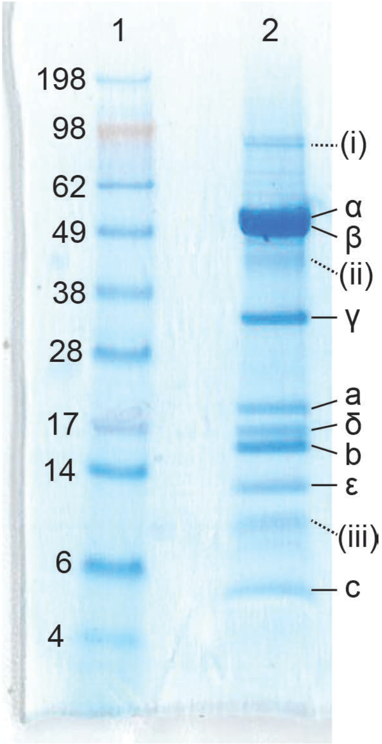

Supplementary Figure 23 SDS PAGE gel showing *E. coli* ATP synthase purity. Lane 1: SeeBlue plus 2 marker (numbers shown are molecular weights in kDa). Lane 2: Purified *E. coli* ATP synthase with subunits bands labelled. Minor contamination bands were identified with mass spectrometry as *E. coli* proteins: i) Ribonuclease E, ii) GroEL and iii) ElaB. We have purified *E. coli* ATP synthase ~50 times, with similar purity obtained each time[9].

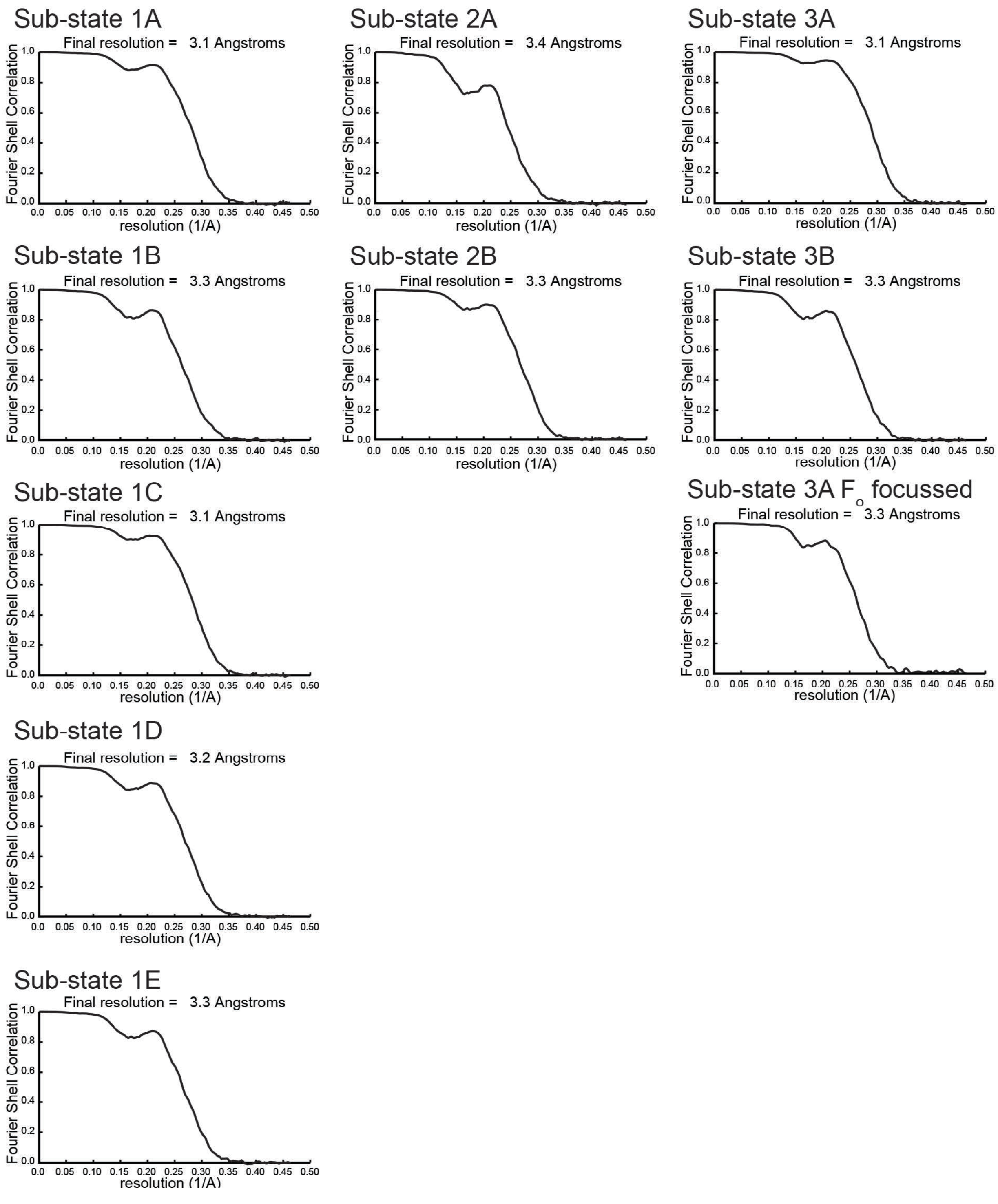

Supplementary Figure 24 Fourier shell correlation curves for states shown in Supplementary Figure 2. Masked FSC curves from Relion[3] postprocess.

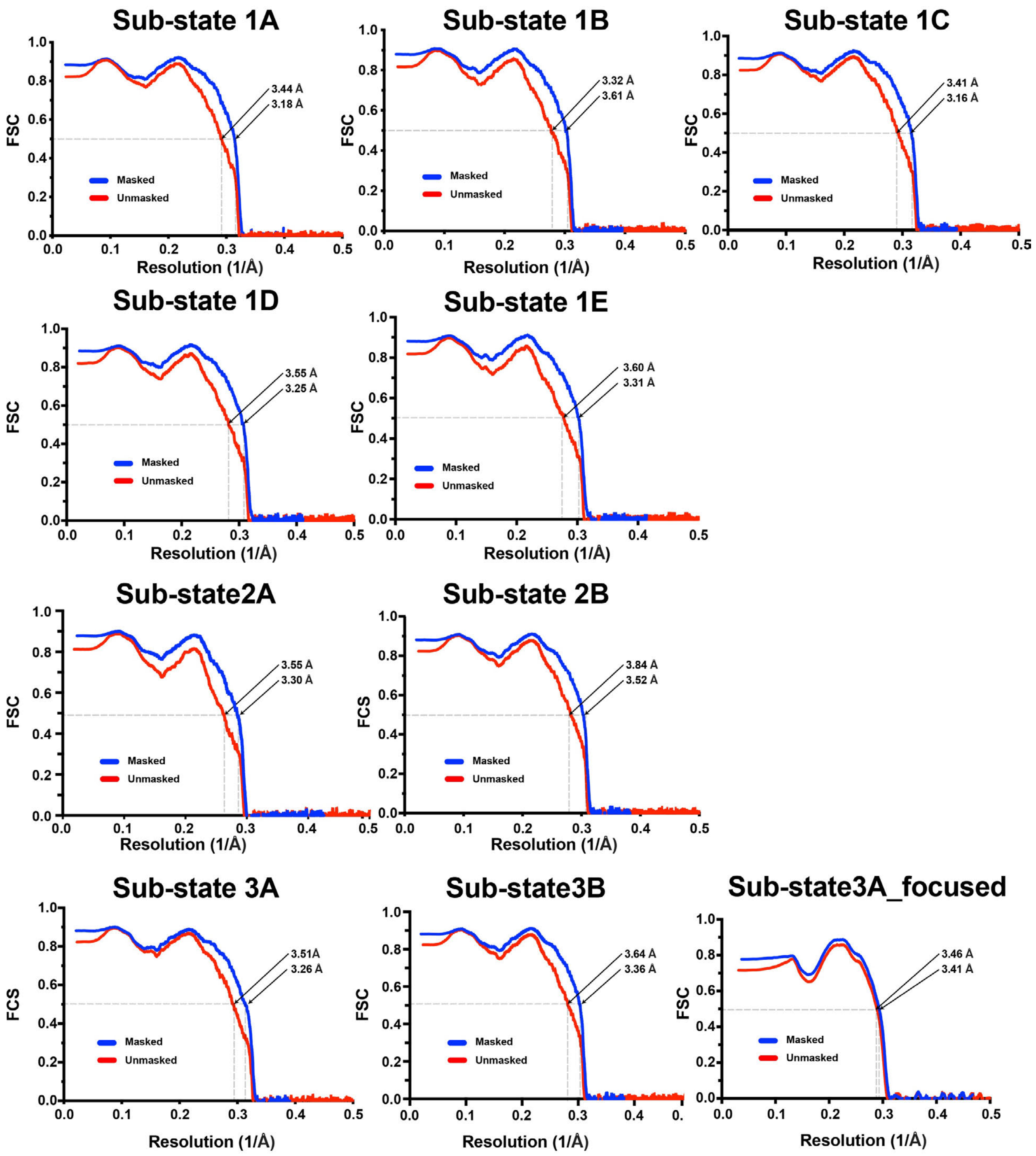

Supplementary Figure 25. FSC map-model curves for the ten deposited structures. Masked and unmasked curves shown in blue and red respectively with resolution estimate at FCS = 0.5 is indicated. Map-model FSC curves generated using phenix.mtriage[6].

Supplementary Table 1 Cryo-EM data collection, refinement and validation statistics.

	#1 Sub-state1A (EMDB-20167) (PDB 6OQR)	#2 Sub-state1B (EMDB-20168) (PDB 6OQS)	#3 Sub-state1C (EMDB-20169) (PDB 6OQT)	#4 Sub-state1D (EMDB-20170) (PDB 6OQU)	#5 Sub-state1E (EMDB-20454) (PDB 6PQV)	#6 Sub-state2A (EMD-21854) (PDB 6WNQ)	#7 Sub-state2B (EMDB-20171) (PDB 6OQV)	#8 Sub-state3A (EMDB-20172) (PDB 6OQW)	#9 Sub-state3B (EMD-21855) (PDB 6WNR)	#10 Sub-state3A F_o Focus (EMDB-21419) (PDB 6VWK)
**Data collection and processing**										
Magnification	81,000	81,000	81,000	81,000	81,000	81,000	81,000	81,000	81,000	81,000
Voltage (kV)	300	300	300	300	300	300	300	300	300	300
Electron exposure ($e^-/Å^2$)	48	48	48	48	48	48	48	48	48	48
Defocus range (μm)	0.8-3.5	0.8-3.5	0.8-3.5	0.8-3.5	0.8-3.5	0.8-3.5	0.8-3.5	0.8-3.5	0.8-3.5	0.8-3.5
Pixel size (Å)	1.08	1.08	1.08	1.08	1.08	1.08	1.08	1.08	1.08	1.08
Symmetry imposed	C1	C1	C1	C1	C1	C1	C1	C1	C1	C1
Initial particle images (no.)	709,190	709,190	709,190	709,190	709,190	709,190	709,190	709,190	709,190	709,190
Final particle images (no.)	52,110	35,243	54,084	33,239	30,404	27,449	61,225	78,283	39,486	78,283
Map resolution (Å) 0.143 FSC threshold	3.1	3.3	3.1	3.2	3.3	3.4	3.3	3.1	3.3	3.3
**Refinement**										
Initial models used (PDB codes)	3oaa, 1abv and 6n2y	3oaa, 1abv and 6n2y	3oaa, 1abv and 6n2y	3oaa, 1abv and 6n2y	3oaa, 1abv and 6n2y	3oaa, 1abv and 6n2y	3oaa, 1abv and 6n2y	3oaa, 1abv and 6n2y	3oaa, 1abv and 6n2y	6n2y
Model resolution (Å) 0.5 FSC threshold, masked/ unmasked	3.2/3.4	3.3/3.6	3.1/3.4	3.3/3.6	3.3/3.6	3.5/3.8	3.3/3.6	3.3/3.5	3.4/3.6	3.1/3.5
Map sharpening *B* factor ($Å^2$)	-54	-58	-50	-42	-44	-54	-64	-62	-63	-85
Model composition										
Non-hydrogen atoms	36951	36971	36971	36971	36971	37024	36971	36902	36935	8423
Protein residues	4849	4851	4851	4851	4851	4857	4851	4847	4851	1133
Ligands	12	12	12	12	12	12	12	12	12	-
B factors ($Å^2$)										
Protein	110	103	91	106	107	103	122	104	93	30
Ligand	81	76	71	80	79	78	99	74	68	-
R.m.s. deviations										
Bond lengths (Å)	0.010	0.007	0.008	0.009	0.009	0.011	0.010	0.005	0.005	0.009
Bond angles (°)	1.195	0.810	0.823	0.886	0.892	1.129	0.897	1.115	0939	1.220
Validation										
MolProbity score	1.72	1.60	1.51	1.80	1.77	1.60	1.90	1.64	1.26	1.02
Clashscore	4.54	5.63	3.76	4.40	4.38	3.83	4.98	4.49	2.18	2.36
Poor rotamers (%)	2.50	1.77	1.98	2.95	2.58	2.19	2.92	1.57	1.44	0.59
Ramachandran plot										
Favored (%)	96.48	97.46	97.13	96.55	96.34	96.99	95.90	96.63	97.17	99.10
Allowed (%)	3.52	2.52	2.87	3.45	3.64	2.97	4.10	3.31	2.81	0.90
Disallowed (%)	0.00	0.02	0.00	0.00	0.02	0.04	0.00	0.06	0.02	0.00

Supplementary References:

1 Sobti, M. *et al.* Cryo-EM structures of the autoinhibited *E. coli* ATP synthase in three rotational states. *Elife* **5**, doi:10.7554/eLife.21598 (2016).

2 Sobti, M. *et al.* Cryo-EM reveals distinct conformations of *E. coli* ATP synthase on exposure to ATP. *Elife* **8**, doi:10.7554/eLife.43864 (2019).

3 Scheres, S. H. RELION: implementation of a Bayesian approach to cryo-EM structure determination. *J Struct Biol* **180**, 519-530, doi:10.1016/j.jsb.2012.09.006 (2012).

4 Bolla, J. R. *et al.* Direct observation of the influence of cardiolipin and antibiotics on lipid II binding to MurJ. *Nat Chem* **10**, 363-371, doi:10.1038/nchem.2919 (2018).

5 Cingolani, G. & Duncan, T. M. Structure of the ATP synthase catalytic complex F_1 from *Escherichia coli* in an autoinhibited conformation. *Nat Struct Mol Biol* **18**, 701-707, doi:10.1038/nsmb.2058 (2011).

6 Afonine, P. V. *et al.* Real-space refinement in PHENIX for cryo-EM and crystallography. *Acta Crystallogr D Struct Biol* **74**, 531-544, doi:10.1107/S2059798318006551 (2018).

7 Guo, H., Suzuki, T. & Rubinstein, J. L. Structure of a bacterial ATP synthase. *Elife* **8**, doi:10.7554/eLife.43128 (2019).

8 Gu, J. *et al.* Cryo-EM structure of the mammalian ATP synthase tetramer bound with inhibitory protein IF1. *Science* **364**, 1068-1075, doi:10.1126/science.aaw4852 (2019).

9 Sobti, M., Ishmukhametov, R. & Stewart, A. G. ATP Synthase: Expression, Purification, and Function. *Methods Mol Biol* **2073**, 73-84, doi:10.1007/978-1-4939-9869-2_5 (2020).
