# Supplementary material for: Cryo-EM structures provide insight into how E. coli F1Fo ATP synthase accommodates symmetry mismatch
Source: Nat Commun. 2020 May 26;11:2615. doi: 10.1038/s41467-020-16387-2 (PMC7251095; doi:10.1038/s41467-020-16387-2)
Supplement: Supplementary file 3 — Description of Additional Supplementary Files [file 41467_2020_16387_MOESM3_ESM.pdf]

Description of Additional Supplementary Files

File Name: Supplementary Movie 1
Description: Morphing between Sub-states 1A-E highlights the structural differences between the classified sub-states. The order of the morphs does not represent the direction of movement and was chosen to highlight differences not describe a progressive movement. (a) side view, (b) rotated 90 degrees relative to a, (c) viewed from the cytoplasm and (d) viewed from the periplasm. Green dot on left to indicate the transition between each sub-state.

File Name: Supplementary Movie 2
Description: Morphing between Sub-state 1', Sub-state 1", Sub-state 3A and Substate 2B shows how the rotor position is related between the states observed. Viewed from the periplasm, with green dot on left to indicate the transition.

File Name: Supplementary Movie 3
Description: Rotation around the Sub-state 3A F_o focused map showing lipid-like density in wheat. Decreasing the map threshold reveals three strong lipid-like volumes corresponding to the proposed lipid bridge.

File Name: Supplementary Movie 4
Description: Animation to show the transition between autoinhibited and active enzyme. The F_1-ATPase is locked in the presence of ADP, with the β_1 subunit half closed against the ε C-terminal domain. In low concentration of nucleotide the β_1 subunit opens. In high concentrations of ATP, the ε C-terminal domain is able to transition to a down state and the β_1 subunit closes to contact the ɣ subunit.
