# Supplementary material for: Cryo-EM structures provide insight into how E. coli F1Fo ATP synthase accommodates symmetry mismatch
Source: Nat Commun. 2020 May 26;11:2615. doi: 10.1038/s41467-020-16387-2 (PMC7251095; doi:10.1038/s41467-020-16387-2)
Supplement: Supplementary file 8 — Reporting Summary [file 41467_2020_16387_MOESM8_ESM.pdf]

nature research

Corresponding author(s): A G Stewart

Last updated by author(s): Apr 24, 2020

# Reporting Summary

Nature Research wishes to improve the reproducibility of the work that we publish. This form provides structure for consistency and transparency in reporting. For further information on Nature Research policies, see Authors & Referees and the Editorial Policy Checklist .

## Statistics

For all statistical analyses, confirm that the following items are present in the figure legend, table legend, main text, or Methods section.

| n/a | Confirmed | |
|---|---|---|
| [ ] | [x] | The exact sample size (*n*) for each experimental group/condition, given as a discrete number and unit of measurement |
| [ ] | [x] | A statement on whether measurements were taken from distinct samples or whether the same sample was measured repeatedly |
| [x] | [ ] | The statistical test(s) used AND whether they are one- or two-sided<br>*Only common tests should be described solely by name; describe more complex techniques in the Methods section.* |
| [x] | [ ] | A description of all covariates tested |
| [x] | [ ] | A description of any assumptions or corrections, such as tests of normality and adjustment for multiple comparisons |
| [x] | [ ] | A full description of the statistical parameters including central tendency (e.g. means) or other basic estimates (e.g. regression coefficient) AND variation (e.g. standard deviation) or associated estimates of uncertainty (e.g. confidence intervals) |
| [x] | [ ] | For null hypothesis testing, the test statistic (e.g. *F*, *t*, *r*) with confidence intervals, effect sizes, degrees of freedom and *P* value noted<br>*Give P values as exact values whenever suitable.* |
| [x] | [ ] | For Bayesian analysis, information on the choice of priors and Markov chain Monte Carlo settings |
| [x] | [ ] | For hierarchical and complex designs, identification of the appropriate level for tests and full reporting of outcomes |
| [x] | [ ] | Estimates of effect sizes (e.g. Cohen's *d*, Pearson's *r*), indicating how they were calculated |

*Our web collection on statistics for biologists contains articles on many of the points above.*

## Software and code

Policy information about availability of computer code

| | |
|---|---|
| Data collection | SerialEM: v3.7 |
| Data analysis | MotionCorr2:v1.2.1, Gctf:v1.06, RELION:v3.0.6, Coot:v0.9, PHENIX:v1.15-3459, ISOLDE:v1.0b3 |

For manuscripts utilizing custom algorithms or software that are central to the research but not yet described in published literature, software must be made available to editors/reviewers. We strongly encourage code deposition in a community repository (e.g. GitHub). See the Nature Research guidelines for submitting code & software for further information.

## Data

Policy information about availability of data

All manuscripts must include a data availability statement. This statement should provide the following information, where applicable:

- Accession codes, unique identifiers, or web links for publicly available datasets
- A list of figures that have associated raw data
- A description of any restrictions on data availability

Maps and models have been deposited in the EMDB and PDB with the following accession codes: Sub-state 1A; EMD-20167 & PDB-6OQR [http://dx.doi.org/10.2210/pdb6OQR/pdb]. Sub-state 1B; EMD-20168 & PDB-6OQS [http://dx.doi.org/10.2210/pdb6OQS/pdb]. Sub-state 1C; EMD-20169 & PDB-6OQT [http://dx.doi.org/10.2210/pdb6OQT/pdb]. Sub-state 1D; EMD-20170 & PDB-6OQU [http://dx.doi.org/10.2210/pdb6OQU/pdb]. Sub-state 1E; EMD-20454 & PDB-6PQV [http://dx.doi.org/10.2210/pdb6PQV/pdb]. Sub-state 2A; EMD-21854 & PDB-6WNQ [http://dx.doi.org/10.2210/pdb6WNQ /pdb]. Sub-state 2B; EMD-20171 & PDB-6OQV [http://dx.doi.org/10.2210/pdb6OQV/pdb]. Sub-state 3A; EMD-20172 & PDB-6OQW [http://dx.doi.org/10.2210/pdb6OQW/pdb]. Sub-state 3B; EMD-21855 & PDB-6WNR [http://dx.doi.org/10.2210/pdb6WNR /pdb]. Sub-state 3A Fo focused: EMD-21419 & PDB-6VWK [http://dx.doi.org/10.2210/pdb6VWK /pdb]. The source data underlying Supplementary Fig 18 are provided as a Source Data file. Other data are available from the corresponding authors upon reasonable request.

# Field-specific reporting

Please select the one below that is the best fit for your research. If you are not sure, read the appropriate sections before making your selection.

[x] Life sciences  [ ] Behavioural & social sciences  [ ] Ecological, evolutionary & environmental sciences

For a reference copy of the document with all sections, see nature.com/documents/nr-reporting-summary-flat.pdf

# Life sciences study design

All studies must disclose on these points even when the disclosure is negative.

| | |
|---|---|
| Sample size | The sample size (8,290 micrographs and 709,190 particles) was chosen to identify the differences between states reliably. This number is general practice in our field (e.g. Murphy, B. J. et al. Rotary substates of mitochondrial ATP synthase reveal the basis of flexible F1-Fo coupling, Science 364 (2019)). During data collection the first 800 images were also processed to investigate the quality of the data, this knowledge was used to estimate that 8,000-9,000 images would be sufficient to solve ~3 Å reconstructions. |
| Data exclusions | Images were excluded based on their CTF parameters (resolution fit to 5 Å or better), this was to ensure only high-quality images were used and that motion correction had been successful. Particles were excluded based on their general appearance and the number of particles in each set. Exclusion criteria were pre-established, in that we would not select "bad" particles or states that refined poorly. This is general practice in our field (e.g. Scheres S.H.W. "RELION: Implementation of a Bayesian approach to cryo-EM structure determination." Journal of Structural Biology 2012 and Scheres S.H.W. "Chapter Six - Processing of Structurally Heterogeneous Cryo-EM Data in RELION." Methods in Enzymology 2016). |
| Replication | As single particle analysis is an averaging method it is not usual to perform replicates, and hence no replication was performed (though the sample was screened many times at 200 kV showing similar results). This is due, in part, to extraordinary demand on 300 kV microscope time and the cost associated with experiments. |
| Randomization | Relion uses a reference free algorithm for 2D classification that starts with all particles in random sets. Randomization, other than this, is not used in this type of study (Scheres S.H.W. "RELION: Implementation of a Bayesian approach to cryo-EM structure determination." Journal of Structural Biology 2012). |
| Blinding | This study was performed on a single protein sample. Blinding of the data would not have been feasible. Furthermore, computer classification was used to identify the sub-states, which addresses the possibility of operator bias. |

# Reporting for specific materials, systems and methods

We require information from authors about some types of materials, experimental systems and methods used in many studies. Here, indicate whether each material, system or method listed is relevant to your study. If you are not sure if a list item applies to your research, read the appropriate section before selecting a response.

## Materials & experimental systems

| n/a | Involved in the study | |
|---|---|---|
| [x] | [ ] | Antibodies |
| [x] | [ ] | Eukaryotic cell lines |
| [x] | [ ] | Palaeontology |
| [x] | [ ] | Animals and other organisms |
| [x] | [ ] | Human research participants |
| [x] | [ ] | Clinical data |

## Methods

| n/a | Involved in the study | |
|---|---|---|
| [x] | [ ] | ChIP-seq |
| [x] | [ ] | Flow cytometry |
| [x] | [ ] | MRI-based neuroimaging |
